# Establishment and validation of an in vitro co-culture model for oral cell lines using human PBMC-derived osteoclasts, osteoblasts, fibroblasts and keratinocytes

**DOI:** 10.1038/s41598-020-73941-0

**Published:** 2020-10-08

**Authors:** Daniel Steller, Alexandra Scheibert, Tabea Sturmheit, Samer G. Hakim

**Affiliations:** 1 Department of Maxillofacial Surgery, University Hospital of Lübeck, Ratzeburger Allee 160, 23562 Lübeck, Germany; 2 Fraunhofer Research Institution for Marine Biotechnology and Cell Technology, Mönkhofer Weg 239 a, 23562 Lübeck, Germany

**Keywords:** Biological techniques, Cell biology, Medical research, Molecular medicine

## Abstract

Indirect co-culture models with osteoclasts including oral cell lines may be influenced by M-CSF and RANKL in the common cell medium. Therefore, we investigated the viability and proliferation of osteoblasts (OB), fibroblasts (FB) and oral keratinocytes (OK) under stratified medium modification and assessed the differentiation of osteoclasts in each co-culture. The impact of M-CSF and RANKL in the common OC co-culture was assessed for OB, FB and OK via MTT assay via DAPI control. The multinuclearity and function of OC were evaluated by light microscopy, DAPI staining, resorption assay and FACS analysis. The PBMC showed the highest differentiation into OC after an incubation period of 7 days. Furthermore, co-culture with OB enhanced the number of differentiated multinucleated OC in comparison with monoculture, whereas co-culture with OK decreased PBMC multinuclearity and OC differentiation. FB did not influence the number of differentiated OC in a co-culture. RANKL and M-CSF reduction had no impact on OC differentiation in co-culture with FB or OB, whereas this medium modification for OK attenuated PBMC multinuclearity and OC differentiation in all approaches. Supplementation of RANKL and M-CSF can be modified for a co-culture of PBMC with FB or OB without disturbing OC differentiation. Thus, pathogenic processes of bone remodelling involving OB, OC, FB and OK in the oral cavity can be investigated thoroughly.

## Introduction

RANKL inhibitors and bisphosphonates are used to treat osteoclast-induced disorders of bone metabolism such as osteoporosis and bone metastases^1,2^. Both substances stop bone turnover via multiple mechanisms, particularly by influencing OC and OB. They act either by preventing differentiation of osteoclasts or by blocking their activity. High drug concentrations result in osteoclast apoptosis, whereas lower concentrations inhibit bone resorption^3^. As a side-effect observed in experimental works, they inhibit proliferation, adhesion and cell migration of osteoblasts, fibroblasts and keratinocytes in a dose-dependent manner^4–6^. These events are associated with the development of the main side effect of these substances–drug-related osteonecrosis of the jaw^7^. Dealing with this side-effect, it is mandatory to understand the intercellular interaction affecting wound healing. For this reason, the establishment and validation of comprehensive models for an in vitro cell co-culture model to investigate osteoclasts and osteoblasts, as well as gingival fibroblasts and oral keratinocytes is essential. Peripheral mononuclear blood cells (PBMC) are a well-studied source for in vitro osteoclast differentiation. They mature to osteoclasts (OC) by cell fusion and lead to multinucleated cells^8^ with a specific function of bone resorption^9^. To obtain the OC character in vitro, the differentiation medium mostly contains M-CSF and RANKL^10,11^. M-CSF is important for the survival of OCs precursor cells, and RANKL particularly supports resorption activity when cultivated on bone or bone-like materials^12^. There are different settings for the simulation of in vivo osteoclastogenesis. Combination of suitable culture conditions, media supplementation and bone or bone-like materials in a co-culture is crucial to simulate the bone environment^13–15^. The aim of this study was to investigate the influence of indirect co-culture cultivation on the proliferation and differentiation of PBMC-derived OC on one side and the viability of OB, FB and OK in this co-culture on the other side.

## Material and methods

This study was performed to establish and validate an indirect co-culture model of osteoclasts (OC) with different oral cell lines. Peripheral blood mononuclear cells (PBMC) were isolated by gradient density centrifugation with Histopaque. With the aim of achieving a large differentiation percentage within a short period of time from thawed PBMC to OC according to a protocol of Marino et al.^16^, we analysed different times for osteoclast generation and their specific activity. Afterwards, the influences of different concentrations of RANKL, M-CSF and culture medium on the viability of intra-oral cell lines in a co-culture approach were tested via MTT assay and DAPI staining.

### Cells and culture conditions

Primary human gingival fibroblasts (FB) and osteoblasts (OB) were purchased from Provitro (Berlin, Germany). Human oral keratinocytes (OK) were purchased from ScienCell (San Diego, USA). The cells were routinely cultured at 37 °C/5% CO_2_ and kept in the recommended growth medium until reaching the required number of cells. Dulbecco’s Modified Eagle Medium for fibroblasts and osteoblasts (DMEM, Thermo Fisher Scientific Inc, Waltham, USA) was supplemented with 10% foetal bovine serum (FBS, Thermo Fisher Scientific Inc, Waltham, USA) and 1% antibiotics (100 U/ml penicillin G, 100 μg/ml streptomycin, Biochrom, Berlin, Germany). Additionally, 1% l-Glutamine (Biochrom, Berlin, Germany) was added to the OB medium. Human oral keratinocytes (OK) were grown in serum-free oral keratinocyte medium (ScienCell, San Diego, USA) or in Dulbecco’s Modified Eagle’s Medium/Nutrient Mixture F-12 Ham (DMEM/F-12, Sigma Aldrich, St. Louis, USA). DMEM/F12 was supplemented with 1% antibiotic solution (100 U/ml penicillin G, 100 μg/ml streptomycin, Biochrom, Berlin, Germany) and 1% or 5% serum-replacement solution (KnockOut serum replacement (KO-SR, Thermo Fisher Scientific Inc, Waltham, USA). Experiments were performed with FB, OB and OK between passages 3 and 10 in triplicate. Trypsin/EDTA solution (Biochrom, Berlin, Germany) was used for passaging monolayer cultures.

### Isolation and cryopreservation of PBMC

**Figure 1:**
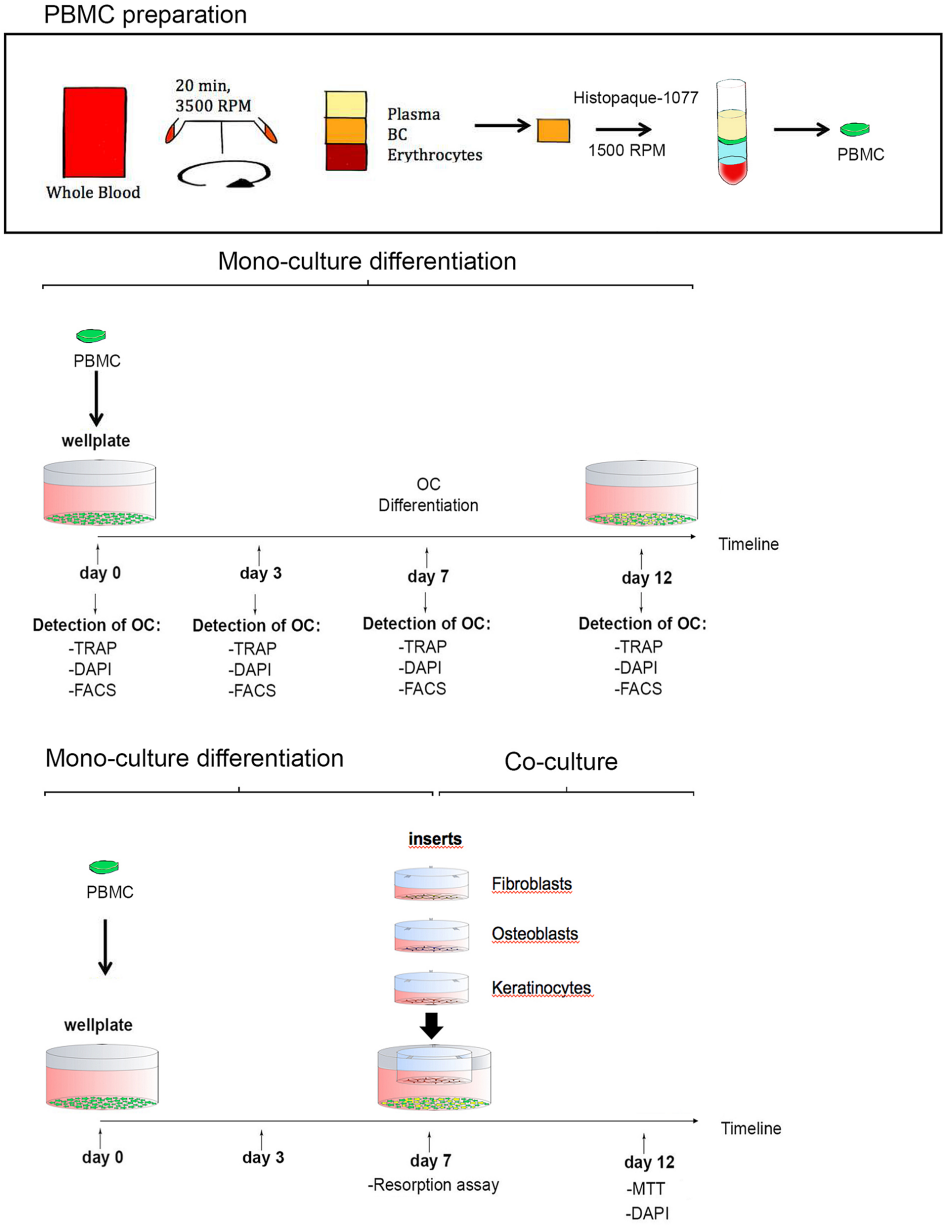
Synopsis illustrating the study design from PBMC isolation to osteoclast differentiation in mono- and co-culture. *PBMC* peripheral blood mononuclear cell, *BC* buffy coat, *OC* osteoclasts, *TRAP*: tartrate-resistant acid phosphatase, *DAPI* 4′,6-diamidino-2′-phenylindole, *FACS* fluorescence-activated cell sorting, *MTT* tetrazolium assay technology.

Whole blood collected from volunteers was provided from the Department of Transfusion Medicine, University hospital of Lübeck. The blood was first centrifuged for 20 min at 22 °C and 3500 RPM to obtain the buffy coat. Buffy coats were transferred into anticoagulant EDTA tube and peripheral blood mononuclear cells (PBMC) were isolated by gradient density centrifugation with Histopaque-1077 (Sigma-Aldrich, St Louis, USA). For this procedure 10 ml of "buffy coat" was mixed with 10 ml of warm (37 °C), sterile phosphate-buffered saline (PBS, Life Technologies, Darmstadt, Germany), layered over 10 ml of Histopaque and centrifuged (800*g*, 30 min, 22 °C, with brake off). The cell layer direct on top of the Histopaque contains PBMC. This layer was transferred into a new 50 ml tube, resuspended in 10 ml of warm PBS, diluted with 10 ml PBS (= 20 ml volume) and centrifuged (300 g, 3 min, 22 °C, with brake on). Subsequently, cells were counted in a haematocytometer, aliquoted, frozen and stored in liquid nitrogen (Fig. 1).

### Osteoclast differentiation of PBMC

Thawed PBMCs were plated in cell-culture dishes at 8.5 × 10^7^ cells per dish in DMEM, 10% FBS, 1% antibiotics, 5% l-Glutamine and 25 ng/ml human M-CSF. After an expansion time of 72 h, the PBMCs were incubated with PBS/EDTA (Life Technologies, Darmstadt, Germany) for 10 min, detached for counting and settled in experimental set up. For osteoclast differentiation in the experimental set-up, medium was supplemented with 25 ng/ml human M-CSF (Peprotech, Rocky Hill, USA) and 50 ng/ml human RANKL (Peprotech, Rocky Hill, USA).

### Validation of osteoclasts via resorption assay and TRAP and DAPI staining

**Figure 2:**
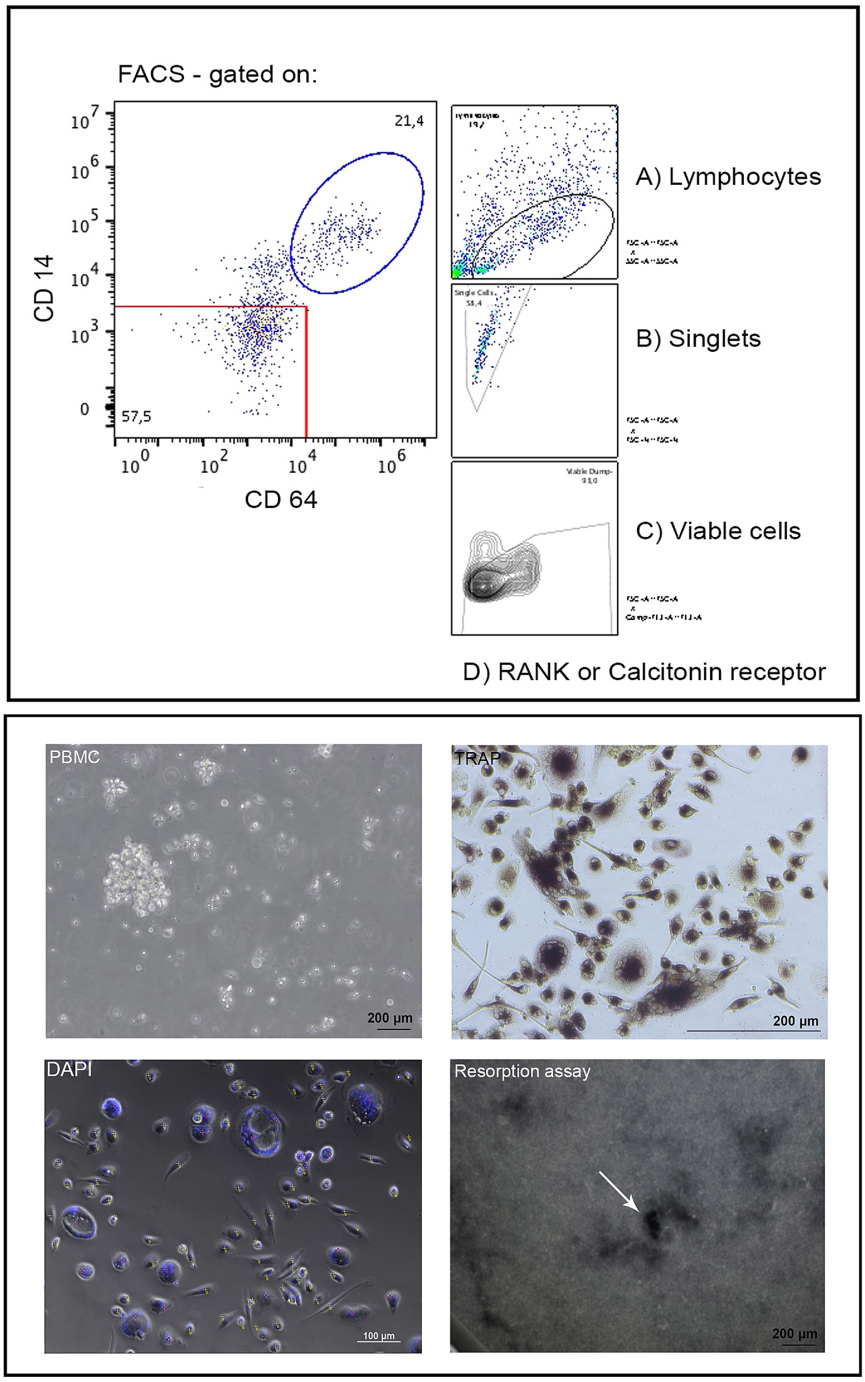
Settings of the gates for FACS analysis are shown in the upper part of the image. In the lower part of the image, representative images of PBMC isolation, TRAP analysis, DAPI stained cell sorting and results of the resorption assay are presented. The white arrow in the lower right image indicates pit formation in the resorption assay. Yellow points in the DAPI staining mark mononuclear cells whereas red dots mark multinuclear ones.

First, DAPI (4′, 6-diamidino-2′-phenylindole, dihydrochloride; Roche, Basel, Switzerland) staining was performed to count the number of nuclei per cell. After rinsing once in PBS, cells were fixed with 100 µl 4% paraformaldehyde (Merck Millipore, Burlington, USA) for 15 min at 22 °C and then incubated with 1 g/ml DAPI in PBS for 5 min. Cell nuclei glow blue when exposed to 345 nm lights (Immunofluorescence microscopy, Axioobserver Z.1, Zeiss, Jena, Germany). Three images were captured per well. These were analysed using the ImageJ software^17^. Only cells containing more than three nuclei were considered multinuclear cells. Second, TRAP activity staining was performed to prove the osteoclast character of the giant cells. The cells were fixed and stained using the acid phosphatase, leukocyte (TRAP) kit (Sigma-Aldrich; Steinheim, Germany) according to the manufacturer’s instructions. Third, the osteoclast bone resorption assay was performed using a commercially available bone resorption assay kit (CosMo Bio, Tokyo, Japan) (Fig. 2). The resorption assay in the mono-culture arm was performed for 7 days only to ensure that the osteoclasts going in co-culture are functionally active cells. The cells were incubated on fluoresceinated calcium phosphate-coated microplate with RANKL (50 ng/ml) in the presence or absence of M-CSF (25 ng/ml). Bone resorption activity was evaluated by detecting the fluorescence intensity of conditioned medium at an excitation wavelength of 485 nm and an emission wavelength of 535 nm.

### Activated cell scanning via flow cytometry of osteoclast marker proteins

Osteoclast differentiation of PBMC induced by M-CSF and RANKL was measured by RANK- and calcitonin-receptor expression via FACS. Multinucleated giant cells of PBMC were identified and tagged by CD3^−^CD19^−^CD14^+^CD64^+^ surface expression^18–20^. Cells were investigated before plastic adhesion (PA) and after 3, 7 and 12 days of osteoclast differentiation. Control and treated cells were harvested and washed once with cold phosphate-buffered saline (PBS). Subsequently, cells were incubated with RANK- and Calcitonin receptor antibody (Human RANK/TNFRSF11A PE-conjugated Antibody and Human Calcitonin R PE-conjugated Antibody; each from bio-techne, RD systems, Minneapolis, United States) in 100 μl PBS at room temperature for 15 min. Cells were washed twice with PBS, and the RANK- and Calcitonin receptor levels were examined by fluorescence-activated cell sorting (FACS; Becton Dickinson, San Jose, CA, USA) (Fig. 2).

### MTT assay for cell proliferation

Recent studies demonstrated that MTT assay provides high sensitivity, reproducibility, stimulation index and a wide linear response range for cell growth in comparison to other methods^21,22^. For MTT assay, viable cells are detected and quantified by mitochondrial conversion of 3-(4,5-dime thylthiazol-2-yl)-2,5-diphenyltetrazoliumbromid (MTT, Sigma Aldrich, St Louis, USA). According to the instructions of the manufacturer, the different cells of the co-culture inserts were set in new wells and rinsed with PBS. Afterwards, 200 μl of MTT dye (1:10 in DMEM/ 10% FBS) was added, incubated for 1 h and rinsed again with PBS. Absorption in the control and experimental groups was measured using a multiwell ELISA reader at a wavelength of 570 nm (Clariostar, BMG-Labtech, Ortenberg, Germany).

### Study design

PBMC were seeded at a density of 8.5 × 10^7^ cells. After a 3-day expansion phase of PBMC, osteoclast differentiation was induced by 50 ng/ml RANKL and 25 ng/ml M-CSF. Differentiation was evaluated for 12 days and controlled by the resorption assay, TRAP and DAPI staining and FACS analysis. After setting the optimal time for the harvest of osteoclasts and the proof of osteoclast function, an indirect co-culture was initiated. A trans-well indirect co-culture system with inserts containing a membrane of 0.4 μm pore size was used to test cell lines separately (ThinCerts cell culture inserts; 0.4 μm pore size, Greiner-bioOne, Kremsmünster, Austria). This model has the advantage to allow exchange of paracrine signaling and response to soluble signalling factors between the upper (insert) and lower chamber of the well. Interaction among cell lines environment remains possible while cells are kept separated for further investigations.

Osteoclasts were seeded in 24-well-plates (Techno Plastic Products, Trasadingen, Switzerland), and the different oral cell lines (fibroblasts, osteoblasts and keratinocytes) were placed in the semipermeable inserts onto the osteoclasts for 5 days (Fig. 1). Co-culture of keratinocytes and PBMC was performed using standard keratinocyte medium or DMEM/F12 (1% or 5% KO-SR) instead of osteoclast medium. Different concentrations of RANKL and M-CSF were tested (RANKL: 50 ng/ml and 25 ng/ml; M-CSF: 25 ng/ml and 12.5 ng/ml) in OB-PBMC and FB-PBMC co-culture but not in OK-PBMC. After 5 days, an MTT assay was performed to investigate the proliferation of osteoblasts, keratinocytes and fibroblasts in co-culture. Further, DAPI staining was performed on the remaining osteoclasts in each co-culture (Fig. 1).

### Statistical evaluation

The experiments were performed in three replicates within each experiment. Statistical evaluation was performed by one-way analysis of variance (ANOVA) applying a post hoc Bonferroni test (IBM SPSS Statistics Version 25, IBM, Armonk, USA). Statistical significance was considered at *p* ≤ 0.05. All data are expressed as the mean ± standard deviation.

### Ethics statement

The local Ethics Committee of the University of Lübeck, Germany approved this study (ID 16-348) and all participants (volunteers) signed informed consent according to the guidelines and regulations of the Declaration of Helsinki.

## Results

### OC differentiation of PBMC in a monoculture

OC differentiation was investigated by obtaining multinucleated cells from PBMC, FACS analysis of RANKL and calcitonin receptor expression, as well as by the functional resorption assay.

#### DAPI staining by light microscopy

**Figure 3:**
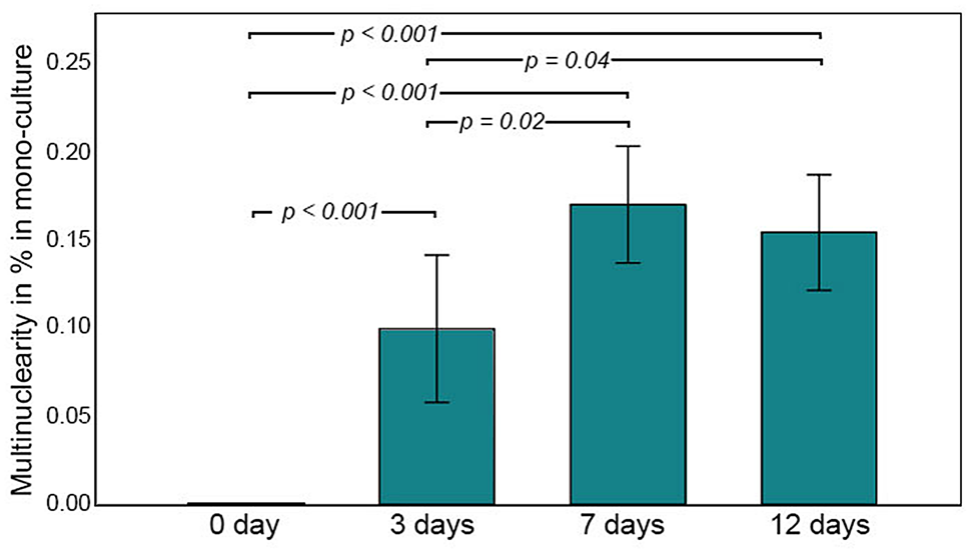
Changes in the percentage of multinuclear PBMC in monoculture in osteoclast standard medium within the observation period of 12 days.

Osteoclast differentiation of PBMC, evaluated as the number of multinucleated cells, increased significantly in number after 7 days of cultivation in comparison to 0 and 3 days (7 days: 17 ± 2% increase, compared to 0 days: 0%; *p* < 0.001; and 3 days: 10 ± 2%; *p* = 0.02). Longer cultivation up to 12 days did not result in further differentiation (12 days: 15 ± 1%; *p* > 0.1; Fig. 3).

#### Differentiation by expression of calcitonin and RANK receptor

**Figure 4:**
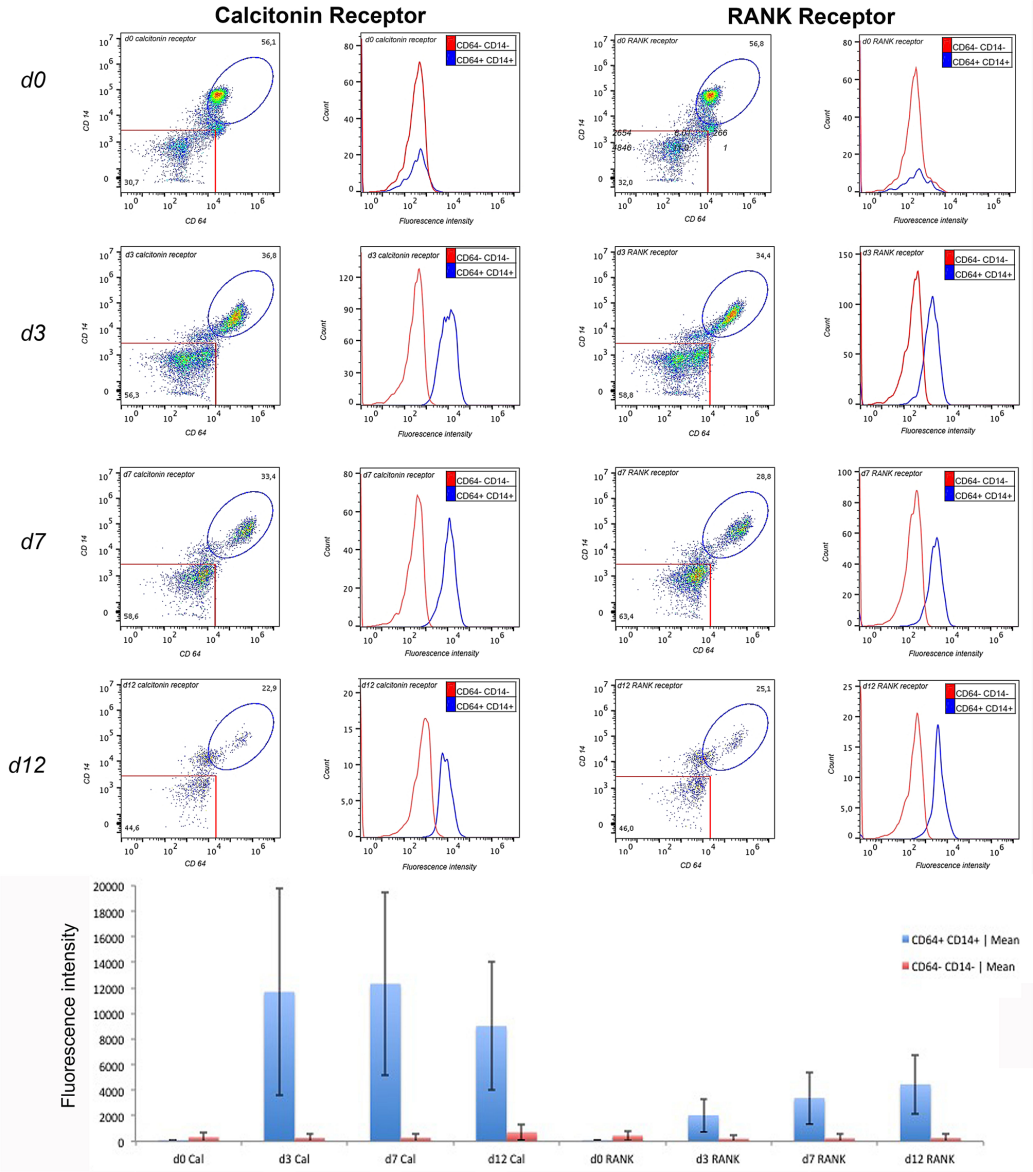
FACS analysis of calcitonin and RANK receptors expression in CD64^+^ and CD14^+^ cells during the differentiation period.

FACS analysis of RANK and calcitonin receptor expression in PBMCs showed an increase in calcitonin receptor expression after 3 days with a maximum at 7 days (mean fluorescence intensity d0: 87 ± 9; d3: 11,699 ± 8096; d7: 12,336 ± 7155; d12: 9024 ± 5004), whereas the expression of RANK receptor showed a slight and continuous increase over this time period (mean of fluorescence intensity d0: 106 ± 4; d3: 2046 ± 1307; d7: 3394 ± 2044; d12: 4467 ± 2329) (Fig. 4).

#### Resorption assay

Considering day 7 as a peak for the multinuclearity and RANK / calcitonin receptor expression in PBMC, we investigated the resorption function of OC at this time. The calcium phosphate (CaP)-coated plates showed distributed pit formation, indicating the resorption capacity of differentiated OC. As a sham-control, only PBMC medium was administered on the (CaP)-coated plates. The use of 25 ng/ml M-CSF and 50 ng/ml RANKL for OC differentiation led to a significant increase in the resorption activity of OC in contrast to RANKL-free M-CSF (mean of fluorescence intensity: M-CSF + RANKL: 2419 ± 64 versus M-CSF: 2227 ± 36; *p* = 0.012).

### OC differentiation of PBMC in the indirect co-culture model

After confirmation of OC differentiation in the monoculture pre-study (day 7), OC were transferred onto the co-culture model. This was then continued in each group for a further 5 days (Fig. 1). This time period was defined according to the pre-study, where RANK- and calcitonin expression had reached a stable peak (Fig. 4).

**Figure 5:**
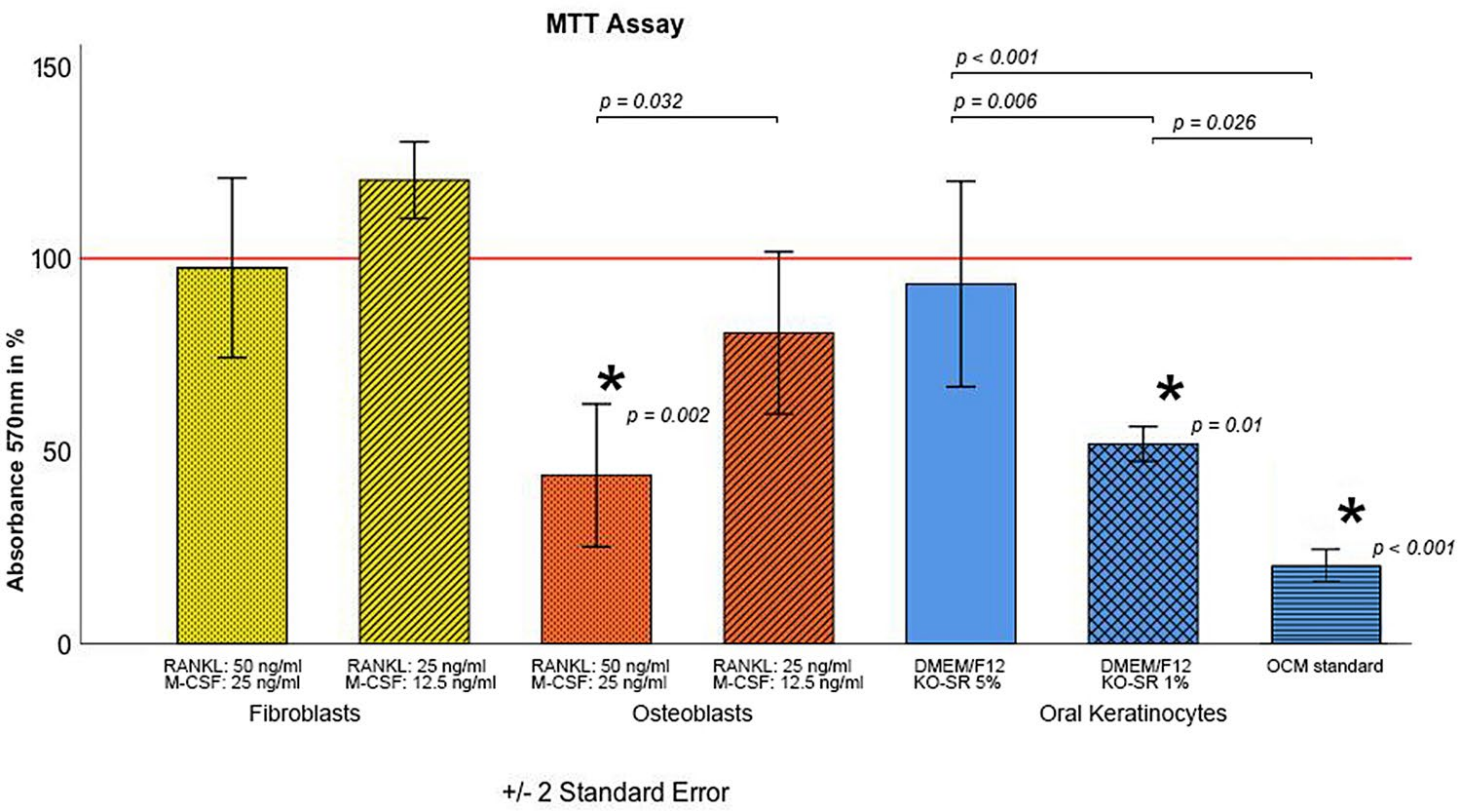
MTT assay indicating viability/proliferation of fibroblasts (yellow), osteoblasts (red) and keratinocytes (blue) under stratified medium modification in co-culture, each with osteoclasts. The horizontal red line represents values for the standard medium of each cell line in monoculture. DMEM/F12: Dulbecco’s modified Eagle’s medium/nutrient mixture F-12 ham; *KO-SR* KnockOut- serum replacement, *OCM* osteoclast standard medium.

**Figure 6:**
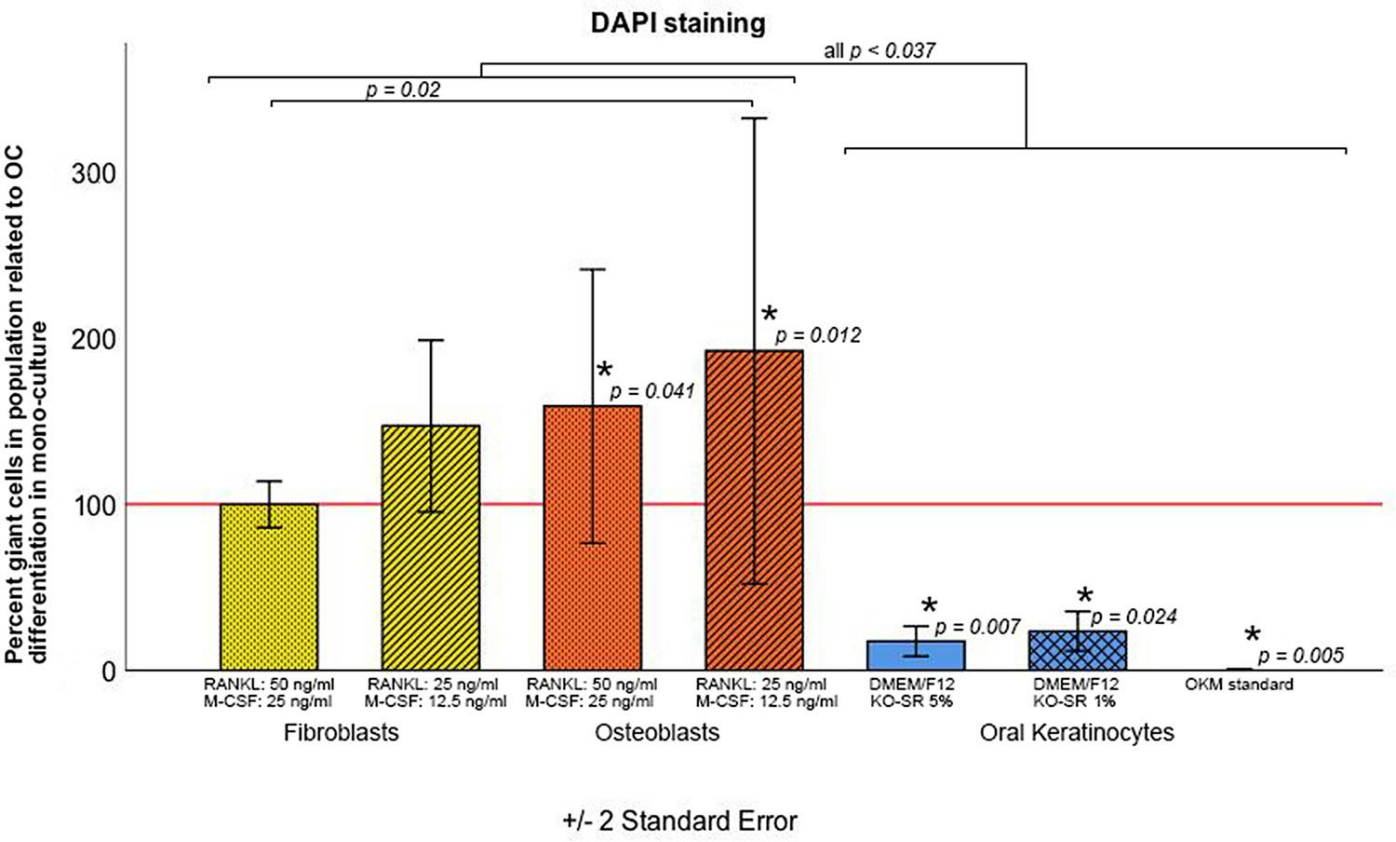
Percentage of multinuclear cells with OC differentiation in the co-culture with OB and OK in relation to mono-culture OC differentiation under stratified medium modification. *DMEM/F12* Dulbecco’s modified Eagle’s medium/nutrient mixture F-12 ham, *KO-SR* KnockOut- serum replacement, *OKM* oral keratinocytes standard medium.

At this time point (day 5 of co-culture), the impact of medium modification on fibroblasts (FB), osteoblasts (OB) and oral keratinocytes (OK) was assessed. For fibroblast co-culture, two different concentrations of RANKL and M-CSF (RANKL = 50 ng/ml and RANKL = 25 ng/ml, as well as M-CSF = 25 ng/ml and M-CSF = 12.5 ng/ml) showed no impact on fibroblast proliferation in comparison with FB medium (FB: 100% ± 0.0%; 25 ng/ml M-CSF and 50 ng/ml RANKL-FB: 97.6 ± 11.7%; 12.5 ng/ml M-CSF and 25 ng/ml RANKL-FB: 120.3 ± 5.7%; both *p* > 0.18; Fig. 5). The reduction of M-CSF and RANKL concentration also did not affect the number of multinucleated cells in the fibroblast-osteoclast co-culture (OC without co-culture: 100% ± 0.0%; 25 ng/ml M-CSF and 50 ng/ml RANKL-FB: 99.9 ± 6.9%; 12.5 ng/ml M-CSF and 25 ng/ml RANKL-FB: 147.0 ± 25.8%; both *p* > 0.21; Fig. 6).

For OB, a common medium with 25 ng/ml M-CSF and 50 ng/ml RANKL led to a significant decrease in cell proliferation in comparison with the lower concentration medium (12.5 ng/ml M-CSF and 25 ng/ml RANKL) and to the standard OB medium (OB: 100% ± 0.0%; 25 ng/ml M-CSF and 50 ng/ml RANKL-OB: 43.7 ± 2.8%; *p* = 0.002; 12.5 ng/ml M-CSF and 25 ng/ml RANKL-OB: 80.7 ± 18.2%; *p* = 0.347; Fig. 5). Looking at the differentiated OC in the co-culture, a significant increase in multinucleated cells was observed in comparison with the monoculture (OC in monoculture: 100% ± 0.0%; 25 ng/ml M-CSF and 50 ng/ml RANKL-OB: 158.9 ± 41.1%; 12.5 ng/ml M-CSF and 25 ng/ml RANKL-OB: 192.1 ± 70.0%; both *p* < 0.041; Fig. 6). Keratinocyte proliferation decreased significantly in osteoclast medium as well as in DMEM/F12-Ko-SR 1% (both *p ≤ *0.01) but was comparable to that in standard medium with DMEM/F12-Ko-SR 5% (*p* = 0.71). Further, the percentage of differentiated OC in the keratinocyte co-culture was significantly low in all conditions compared to those in the monoculture (*p* < 0.02). DMEM/F12 provided higher OC differentiation than the OKM standard medium.

## Discussion

Osteoclasts are a crucial cell type in bone remodelling. They are the main target of bisphosphonates and RANKL inhibitors in patients treated for osteoporosis, bone metastases and bone resorption-induced hypercalcemia. Administration of these drugs leads, therefore, to disturbed bone turnover and subsequent necrosis of the jaw^1,2^. In the current study we established an indirect co-culture model to investigate contactless cell–cell interaction between PBMC-derived osteoclasts and the main oral cell lines in vitro. Thus, the effect of soluble substances and media on viability and proliferation of osteoblasts, fibroblasts and keratinocytes can be better studied and understood.

### OC differentiation from PBMC in co-culture with OB, FB and OK

Several protocols have been reported for osteoclast differentiation from PBMC in vitro^16,23,24^. These protocols are often time-consuming and incapable of generating sufficient numbers of osteoclasts^10^. Bernhardt et al. compared different PBMC isolation methods for obtaining a homogenous cell population and for improving osteoclast generation. They concluded that simple density gradient centrifugation, as performed in the current study, leads to optimal osteoclast differentiation between 9 and 16 days^25^. In this study, PBMCs were differentiated to osteoclasts by adding 25 ng/ml M-CSF and 50 ng/ml RANKL to the standard medium. Using this method, we could show that PBMC had the highest number of multinucleated cells after 7 days. A longer cultivation period did not improve OC differentiation. We assessed the advantage of RANKL supplementation to induce active osteoclasts in contrast to M-CSF alone. In accordance with our results, Cody et al. showed high OC differentiation after 7–8 days by adding a lower concentration of M-CSF to the standard medium^10^. Similarly, they observed more efficient OC formation in medium supplemented with 50 ng/ml RANKL compared with 10 ng/ml, whereas increasing the concentration to 100 ng/ml did not increase the osteoclast yield further^10^. In contrast to these findings, Costa-Rodriguez et al. showed that long-term supplementation—up to 21 days—with conditioned media derived from supernatants from either fibroblast or osteoblast cell cultures stimulated osteoclastogenesis of PBMC without addition of M-CSF and RANKL, since both cultures used to prepare the conditioned media express RANKL and M-CSF themselves^26^. In the present study, we also observed an impact of osteoblasts and fibroblasts on osteoclastogenesis of PBMC. Using 12.5 ng/ml M-CSF and 25 ng/ml RANKL in the indirect co-culture of PBMC and osteoblasts led to significant enhancement of osteoclast differentiation in relation to PBMC monoculture, although this effect was higher in osteoblast co-culture. Based on these findings, it becomes clear that a reduction of M-CSF and RANKL concentration in indirect co-culture of PBMC with osteoblasts and fibroblasts does not attenuate osteoclast differentiation and activity. The decrease of OC differentiation from PBMC in OK medium observed in the present study can be explained by the missing RANKL and M-CSF stimulus and the absence of FBS in the culture medium^23^. In order to overcome this problem, Iwamoto et al. cultivated keratinocytes and fibroblasts together with OC and could show sufficient RANKL expression to differentiate osteoclasts in a co-culture model. Intercellular communication among the different cell lines involved in the co-culture seems to trigger local RANKL expression^27^.

### Proliferation of OB, FB and OK in co-culture with OC

The proliferation of the osteoblasts was compromised using medium with 25 ng/ml M-CSF and 50 ng/ml RANKL, while this effect was not evident at lower M-CSF and RANKL concentrations (12.5 ng/ml and 25 ng/ml, respectively). The intervention in the RANKL/OPG ratio might be a reason for this observed impact on OB proliferation. It is known that RANKL up-regulation and OPG down-regulation lead to bone loss via several endogenous factors that regulate the RANKL/RANK/OPG pathway, including cytokines and mesenchymal transcription factors^28,29^. The establishment of an indirect keratinocyte-PBMC co-culture seems to represent a challenging issue, as we could not keep the OK and OC in co-culture viable or differentiated for the initial 5 days. Both cell lines underwent significant decreases in proliferation and differentiation in both media. The proliferation loss of OK in OC medium supports the assumption by Shipley et al., who reported on the advantage of serum-free medium for in vitro culturing of keratinocytes^30^. Compared with the standard medium of each cell line, co-culturing using DMEM/F12 with 5% of knock-out serum replacement solution, however, results in a high proliferation rate of OK along with a relatively reasonable OC differentiation.

## Conclusion

We show that OC differentiation of PBMC could be reached after 7 days in monoculture. Both OB and FB enhance PBMC differentiation onto OC in an indirect co-culture model and thus allow reduction of M-CSF and RANKL concentration in the medium without impact on OC differentiation.

The results presented above contribute to the establishment of an indirect co-culture model for the investigation of environmental and soluble factors, which influence bone remodelling in pathogenic processes of the oral cavity, where OB, OC, FB and OK interact. The mechanism underlying altered osteoclasts differentiation in co-culture is an interesting and relevant aspect from the physiologic point of view, that has not been investigated in the current study but may be addressed in future studies to better develop therapeutic approaches.
